# Changes in the Phenolic Compounds of Hop (*Humulus lupulus* L.) Induced by Infection with *Verticillium nonalfalfae*, the Causal Agent of Hop *Verticillium* Wilt

**DOI:** 10.3390/plants9070841

**Published:** 2020-07-03

**Authors:** Urban Kunej, Maja Mikulič-Petkovšek, Sebastjan Radišek, Nataša Štajner

**Affiliations:** 1 Department of Agronomy, Biotechnical Faculty, University of Ljubljana, 1000 Ljubljana, Slovenia; urban.kunej@bf.uni-lj.si (U.K.); maja.mikulic-petkovsek@bf.uni-lj.si (M.M.-P.); 2 Plant Protection Department, Slovenian Institute of Hop Research and Brewing, 3310 Žalec, Slovenia; sebastjan.radisek@ihps.si

**Keywords:** *Humulus lupulus*, *Verticillium nonalfalfae*, fungal infection, phenolic compounds, plant–pathogen interactions

## Abstract

Phenolic compounds are involved in plant responses to various biotic and abiotic stress factors, with many studies suggesting their role in defense mechanisms against fungal pathogens. Soilborne vascular pathogen *Verticillium nonalfalfae* causes severe wilting and consequent dieback in a wide range of economically important crops, including hops (*Humulus lupulus* L.). In this study, we investigated the differential accumulation of phenolics in the susceptible “Celeia” and resistant “Wye Target” hop cultivars during the pathogenesis of *Verticillium* wilt. Quantitative polymerase chain reaction showed that colonization in the roots of both cultivars was intensive, but decreased continuously throughout the experiment in the resistant cultivar, while the relative fungal amount continuously increased in the stems of the susceptible cultivar. In response to colonization in the roots of the resistant cultivar, a significant increase in total flavanols was detected at three days postinoculation (dpi), suggesting a possible role in preventing fungus spread into the stems. The accumulation of phenolic compounds was less pronounced in the stems of the resistant cultivar since, compared to the latter, significant increases in flavonols at 3 and 15 dpi and hydroxycinnamic acids at 6 dpi were observed in the stems of the susceptible cultivar.

## 1. Introduction

Hops (*Humulus lupulus* L.) is a plant native to Europe, North America, and Western Asia. It is grown commercially for use in the brewing industry as an essential ingredient that adds flavor, and as a stabilizer or preserver [1]. Due to its importance in the brewing industry, its cultivation has spread to South Africa, Australia, and New Zealand [2]. According to the International Hop Growers’ Convention (IHGC), global hop cultivation was over 61,781 hectares (ha), producing 122,767 metric tons (Mt) of hops in total [3].

Some major factors that limit the production of hops in many hop-producing areas are caused by soilborne pathogens, such as *Verticillium nonalfalfae* (Inderb., H. W. Platt, R. M. Bostock, R. M. Davis and K. V. Subbarao; formerly known as *Verticillium albo-atrum*) and *Verticillium dahliae* (Kleb.). Symptoms of the disease caused by *V. nonalfalfae* vary depending on the aggressiveness of the strain and the sensitivity of the cultivars. In general, two disease forms are recognized in hops, i.e., mild and lethal wilt. Mild wilt arises from infections of sensitive hop cultivars by the mild pathotype of *V. nonalfalfae*. Symptoms vary in intensity from season to season and rarely end in plant death. Highly virulent (lethal) pathotypes of *V. nonalfalfae* are causal agents of lethal wilt in susceptible varieties that develop severe leaf chlorosis, necrosis, and dieback of the rootstock within a few months after infection [4]. 

Infection of a host by *V. nonalfalfae* is initiated by the germination of dormant structures that are activated by plant-root exudates. Afterward, hyphae penetrate the epidermal cells of the root, enter the vascular system, and start to form conidiospores that systematically spread throughout the entire plant by xylem-sap flow in xylem vessels [5]. During colonization, pathogens produce and secrete hydrolytic enzymes that are involved in the degradation of the plant cell wall, thus facilitating the spread of the fungus [6,7]. *V. nonalfalfae* is very difficult to control due to ineffective fungicides that do not suppress fungus colonization in plants, and its resting structures that are viable in the soil for many years. Therefore, the most efficient methods for disease management are breeding and planting resistant varieties, crop rotation, and other soil-disinfestation methods [8]. However, due to the low efficacy of currently used methods, new strategies for *Verticillium* wilt management are needed. Understanding the mechanisms of resistance and immune response to fungal infection is one of the most important means of plant-disease management. 

Phenolics are strongly involved in plant–pathogen interactions and may restrict the spread of a pathogen [9]. Plant phenolics are secondary metabolites consisting of structurally diverse compounds arising from shikimate–phenylpropanoid pathways. Upon recognition of pathogens, plant defense can be either constitutive, e.g., formation of structural barriers and phytoanticipins (compounds that occur constitutively) [10], or induced, e.g., synthesis of polyphenols and cell-wall fortification, and the production of reactive oxygen species (ROS), pathogenesis-related proteins, and phytoalexins (inducible secondary metabolites) [11,12]. During the pathogenesis of fungal diseases, the content and composition of polyphenolics changes, thereby inducing disease resistance [13,14,15,16,17,18]. Plants were shown to respond to fungal invasion by accumulating phenolics and phenolic polymers, such as lignin, flavanols, ferulic acid, *p*-coumaric acid, and sinapic acid in the cell wall, thus inhibiting pathogen enzymes that degrade cell walls [19,20]. 

Most previous studies investigating the response of hops to *V. nonalfalfae* were generally descriptive and did not report the phenolic response or composition of hop phenolics during the pathogenesis of *Verticillium* wilt. Only a few studies reported on the biochemical and physiological response of hop plants to infection with *V. nonalfalfae*. For example, susceptible and resistant hop cultivars respond to infection by a lethal *V. nonalfalfae* pathotype with the secretion of gelatinous and gum compounds that restrict the spread of the fungus [21]. Additionally, the induced amounts of sugars and starches in root tissue invaded by *V. nonalfalfae* inhibit the secretion of cellulase, thus preventing the destruction of the cell wall and invasion of the pathogen [22], but defensive reactions reported in the roots of the susceptible cultivar were not effective and could not eliminate the pathogen from the vascular system, resulting in invasion of the hyaline fungal mycelium [23]. After the elicitation of hop cell cultures with *V. nonalfalfae* culture filtrate, cell cultures of resistant cultivar showed two- to threefold higher activity of phenylalanine ammonia-lyase (PAL), which is the first enzyme in the phenylpropanoid pathway [24,25]. 

Studies investigating the interaction between *Verticillium dahliae* and olives showed that the infection affects the accumulation of volatile compounds and phenolics in the oil [26]. When studying the response of resistant and susceptible olive cultivars to defoliating and nondefoliating *V. dahliae* pathotypes in vascular tissue, Markakis et al. [27] found a significantly higher amount of verbascoside in the resistant olive cultivar, a compound that demonstrates antimicrobial activity. In both cultivars, the contents of total phenols and oleuropein were negatively correlated with the relative amount of fungal DNA. Eynck et al. [16] investigated the differential accumulation of phenolic compounds in susceptible and resistant cultivars of winter oilseed rape inoculated with *Verticillium longisporum* and observed an intensive phenolic response in the resistant genotype, whereby accumulation of cell-wall-bound phenolics was observed during the early stages of the infection, and lignin deposition in later stages. In potato, El-Bebany et al. [18] showed that infection with *V. dahliae* causes differential accumulation of hydroxycinnamic acids, especially ferulic and caffeic acid, in the roots or stems, and accumulation was higher in tissues inoculated with a highly aggressive isolate of *V. dahliae*. The stem tissue inoculated with the highly aggressive isolate showed lower rutin accumulation compared to stems inoculated with a mild isolate. 

The quantification and composition of phenolic compounds during fungal infection of plants are of crucial importance due to their proposed roles in plant defense. To date, resistance mechanisms based on the spatial and temporal dynamics of phenolic compound contents during infection with *V. nonalfalfae* are not known in hop plants. Therefore, in this study, changes in the content of individual phenolic compounds and their involvement in the development of a plant defense were evaluated. Plant samples were chosen on the basis of the fungal colonization profiles evaluated in this study. To our knowledge, this is the first study describing detailed compositions of phenolic profiles and contents of individual phenolic compounds in resistant and susceptible hop cultivars during the pathogenesis of *Verticillium* wilt caused by *V. nonalfalfae*.

## 2. Results

### 2.1. V. nonalfalfae Colonization Profile Analysis in Susceptible and Resistant Hop Cultivars

Colonization profiles and estimation of the relative amount of fungi in plants after infection are essential to further evaluate plant response mechanisms during pathogenesis. Colonization in the samples was evaluated to accurately assess the relative amount of fungal DNA in each sample, i.e., to determine biological replicates that were similar in terms of fungal spread and potentially at the same stage of infection.

**Figure 1:**
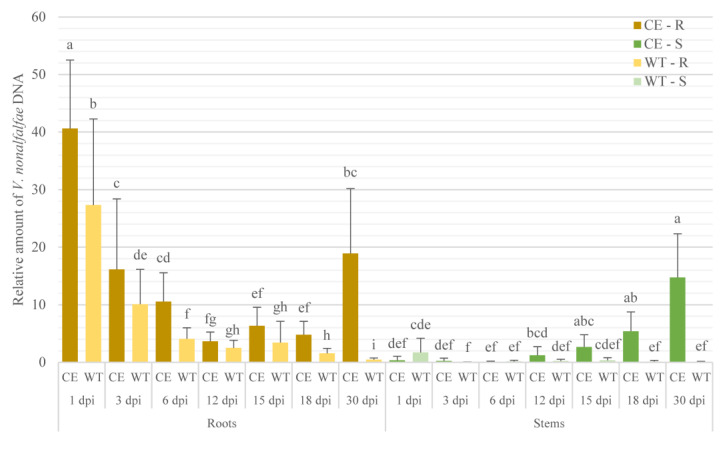
Colonization of *V. nonalfalfae* in roots (R) and stems (S) of susceptible (“Celeia”, CE) and resistant (“Wye Target”, WT) hop cultivars at different sampling times after inoculation (days postinoculation, dpi). Fungus quantity was determined with qPCR and normalized to hop reference gene DRH1. Data were analyzed by one-way ANOVA and Duncan’s post hoc test for root samples, and by the Kruskal–Wallis rank-sum test and the Holm–Bonferroni method for stem samples. Different letters denote statistically significant differences between root or stem samples at *p* ≤ 0.05. Data are presented as mean ± SD.

According to our data, we observed high standard deviation of the biological replicates within the same sampling time, as shown in Figure 1, which had the potential to obscure plant defense responses during infection in further analyses. On the basis of these results, we only used samples with similar amounts of fungi for further analysis of the phenolic compounds.

In the roots of the susceptible cultivar “Celeia” (CE-R), the relative amount of *V. nonalfalfae* DNA was observed in a bimodal pattern, with peaks at 1 and 30 days postinoculation (dpi). The highest amount of fungal DNA was detected at 1 dpi, followed by a significant 2.5-fold decrease to 3 dpi, from where it steadily decreased until day 12. At 30 dpi, compared to 18 dpi, a significant 3.9-fold increase in the amount of fungal DNA was detected (Figure 1).

In the stems of the susceptible cultivar Celeia (CE-S), the relative amount of fungal DNA gradually increased throughout the experiment. The highest relative amount of fungal DNA was observed at 30 dpi (14.7-fold), which was significantly higher than at 1 (0.34-fold), 3 (0.23-fold), 6 (0.9-fold), and 12 dpi (1.2-fold). In the susceptible cultivar, the transition of *V. nonalfalfae* from roots to stems appeared at 12 dpi, since roots (3.6-fold) and stems (1.2-fold) showed similar relative fungal DNA amounts (Figure 1). 

Colonization was significantly less extensive in the resistant cultivar “Wye Target” (WT-R) than it was in the susceptible cultivar Celeia (CE-R). In the roots of the resistant cultivar, the highest quantity of fungal DNA was detected at 1 dpi (27.3-fold), which was 1.5-fold lower compared to the susceptible cultivar at the same sampling time (40.6-fold). From 1 to 3 dpi, a significant 2.7-fold decrease was detected, followed by a gradual decrease until 30 dpi. Fungal DNA was also detected in the stems of the resistant cultivar (WT-S), but at very low levels at all sampling times (Figure 1). In control samples or mock-inoculated plants, no fungal DNA was detected.

The colonization profiles thus showed rapid fungal colonization in the roots of both cultivars at 1 dpi and a continuous decrease in relative fungal quantity in the susceptible cultivar, probably due to the spread of the fungus from roots to stems.

### 2.2. Phenolic Compounds

Eight phenolic compounds were identified in the roots of both cultivars, all belonging to flavanols (Tables S1 and S1.1); no phenolics belonging to flavonols or hydroxycinnamic acids were detected. 

**Figure 2:**
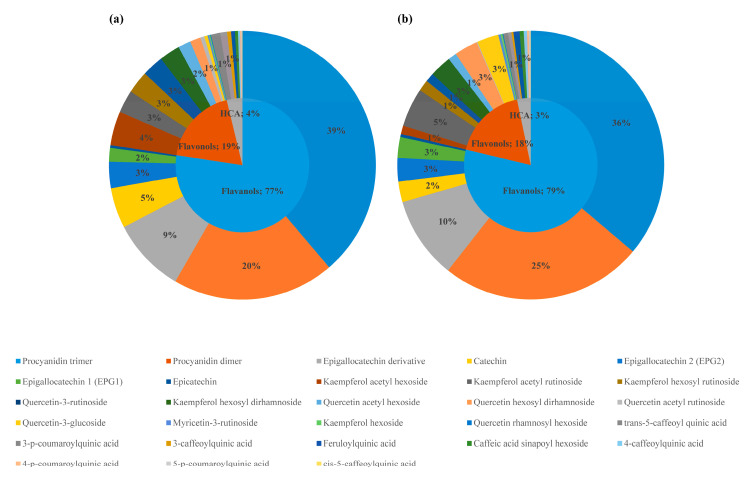
Representation (%) of main phenolic classes (flavanols, flavonols, and hydroxycinnamic acids (HCAs) and individual phenolic compounds determined in stems of (**a**) susceptible and (**b**) resistant hop cultivars.

In the stems of both cultivars, 28 different phenolic compounds were identified that were classified into the phenolic classes of flavanols, flavonol derivatives (flavonols), and hydroxycinnamic acids. In the stems, the most represented among all identified phenolic compounds (IPCs) were flavonols (12 IPCs), followed by hydroxycinnamic acids (9 IPCs), and flavanols (7 IPCs). However, on average, flavanols represented 77% and 79%, flavonols 19% and 18%, and hydroxycinnamic acids (HCAs) 4% and 3% of the total analyzed phenolic compounds (TAPs) in the stem tissue of susceptible and resistant cultivars, respectively (Figure 2).

#### 2.2.1. Flavanols

**Figure 3:**
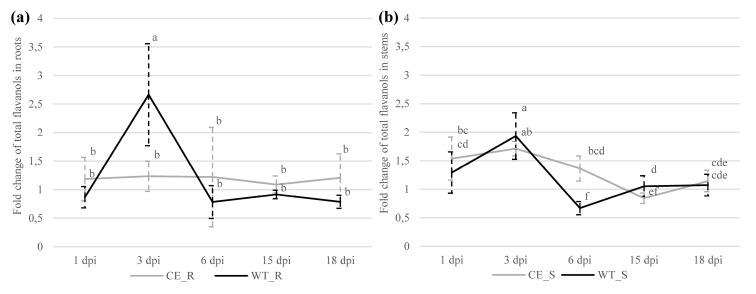
Fold changes (infected/control) of total analyzed flavanols in (**a**) roots (R) and (**b**) stems (S) of susceptible (Celeia, CE) and resistant (Wye Target, WT) hop cultivars. Data are presented as mean ± SD. Values below 1 indicate decreases in content compared to the control. Data were analyzed by one-way ANOVA and Duncan’s post hoc test for root and stem tissues, respectively. Different letters denote statistically significant differences at *p* ≤ 0.05.

In the roots of the susceptible cultivar, no differences in the contents of individual phenolic compounds or total analyzed flavanols were determined between the control and infected root samples at any sampling time. However, in the resistant cultivar, a significant 2.7-fold increase in total analyzed flavanols was detected at 3 dpi in the infected compared to the control root samples (Table S1). After normalizing the contents of total analyzed flavanols to the corresponding controls of each cultivar, a significant 2.2-fold increase was detected in the resistant compared to the susceptible cultivar (Table S1.1). In addition, in the roots of the resistant cultivar, we observed a significant threefold increase in total analyzed flavanols from 1 to 3 dpi (Figure 3a). 

In the stems of infected susceptible cultivar, a significantly higher content of total analyzed flavanols was detected at 1 (1.5-fold) and 3 dpi (1.7-fold); in the resistant cultivar, a significantly higher content was determined only at 3 dpi (1.9-fold) when compared to the control. In stems of the susceptible cultivar, a steady 2-fold decrease in flavanol content was detected from 3 to 15 dpi; in the stems of the resistant cultivar, a strong significant decrease was observed from 3 to 6 dpi (2.9-fold), followed by a significant 1.6-fold increase from 6 to 15 dpi. Total flavanol content significantly decreased at 6 dpi in the stems of the resistant cultivar compared to the stems of the susceptible cultivar (Figure 3b).

Considering individual phenolic compounds in the roots of the resistant cultivar, a significantly higher amount of epigallocatechin 2 (EPG2; 2.4-fold) was detected in the infected compared to the control samples at 3 dpi (Table S1), while a significant 3.5-fold increase of EPG2 was also detected from 1 to 3 dpi (Table S1.1). Furthermore, a significant 2.4-fold increase of EPG2 was observed in the roots of the resistant compared to the susceptible cultivar at 3 dpi (Table S1.1). In the stems, we detected significantly higher amounts of EPG2 in the infected samples of the resistant (2.1-fold) and susceptible (1.7-fold) cultivars at 3 dpi (Table S1); we also observed a significant increase from 1 to 3 dpi in the resistant (1.5-fold) and susceptible (1.4-fold) cultivars (Table S1.1). At 15 dpi, we detected a significantly lower (1.3-fold), and at 18 dpi a significantly higher (1.9-fold), amount of EPG2 in the infected compared to the control stem tissues of the susceptible cultivar (Table S1). Consequently, a significant decrease was detected from 6 to 15 dpi, followed by a significant increase from 15 to 18 dpi (Table S1.1).

In response to infection in the stems, we detected a significantly higher amount of epigallocatechin derivatives in the susceptible cultivar at 1 (1.6-), 3 (2-fold), 6 (1.7-fold), and 18 (1.7-fold) dpi, and in the infected stem samples of the resistant cultivar at 1 (1.7-fold) and 3 dpi (3-fold) (Table S1). In the resistant cultivar, we detected a significant increase in epigallocatechin derivatives from 1 (1.7-fold) to 3 dpi (2.964-fold), which was also significantly higher compared to that of the susceptible cultivar (twofold) at 3 dpi (Table S1.1).

The amount of procyanidin dimer was higher in the infected stem tissue of the susceptible cultivar at 1 (1.8-fold), 3 (1.7-fold), and 6 dpi (1.4-fold). In the infected stem tissue of the resistant cultivar, the amount of procyanidin dimer was higher at 3 dpi (1.7-fold) (Table S1), resulting in a significant increase from 1 to 3 dpi (Table S1.1). At 6 dpi, we detected a significantly lower amount of procyanidin dimer in the infected compared to the control stem tissue of the resistant cultivar (1.7-fold), and a significant decrease compared to the susceptible cultivar (Table S1.1). 

Epigallocatechin 1 (EPG1) was detected in the stem, but not the root, tissues of both cultivars. The amount of EPG1 at 15 dpi was significantly lower (2.1-fold) in the infected compared to in the control stem tissue of the susceptible cultivar; we also detected a significantly higher (1.8-fold) amount in the infected stem tissue of the resistant cultivar. The latter resulted in a significant increase of EPG1 in the resistant compared to in the susceptible cultivar (Table S1.1). At 18 dpi, we detected a significantly higher amount of EPG1 in the infected compared to the control stem tissue in the susceptible (1.6-fold) and resistant (twofold) cultivars (Table S1).

In the stem tissue, procyanidin trimers and dimers represented the greatest portion of TAP in the susceptible (39% and 20%) and the resistant cultivars (36% and 25%), followed by epigallocatechin derivatives (9% and 10% per cultivar, respectively). Catechin represented 5% and 3%, EPG2 3%, and EPG1 2% of TAP in the susceptible and resistant cultivars, respectively (Figure 2).

The strongest response to infection in the stem tissue was observed at 3 dpi, as significant increases in EPG2 (1.7- and 2.1-fold), epigallocatechin derivative (2- and 2.9-fold), procyanidin dimer (1.7-fold, respectively), and procyanidin trimer (1.6- and 1.8-fold) were detected in the infected stem samples of the susceptible or resistant cultivar compared to the corresponding controls. Additionally, significant increases in EPG1 (2.2-fold) and epicatechin (1.6-fold) were only detected in the susceptible cultivar, while a significant increase in catechin (2.1-fold) was only observed in the resistant cultivar (Table S1).

Considering the temporal dynamics of the identified flavanols in the stems from 1 to 3 dpi, significant increases in EPG2 (1.4-fold) and epicatechin (1.9-fold) were detected in the susceptible cultivar, and significant increases in EPG2 (1.5-fold), epigallocatechin derivative (1.8-fold), and procyanidin dimer (1.65-fold) were observed in the resistant cultivar. In the resistant cultivar, all identified flavanols significantly decreased at 6 dpi compared to a prior sampling time (Table S1.1). In the susceptible cultivar, however, decreases in catechin, EPG2, EPG1, and procyanidin dimer and trimer were observed at 15 dpi (Table S1.1).

#### 2.2.2. Flavonol Derivatives

**Figure 4:**
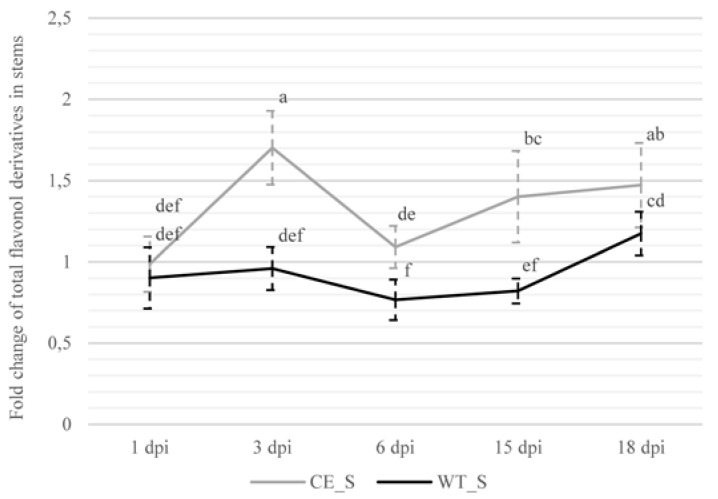
Fold changes (infected/control) of total analyzed flavonols in stems (S) of susceptible (CE; Celeia) and resistant (WT; Wye Target) hop cultivars. Data are presented as mean ± SD. Values below 1 indicate decreases in content compared to the control. Data were analyzed by one-way ANOVA and Duncan’s post hoc test. Different letters denote statistically significant differences at *p* ≤ 0.05.

The amount of total analyzed flavonol derivatives (flavonols) showed a bimodal pattern of accumulation in the infected stem samples of the susceptible cultivar, in which we detected significantly higher amounts of total analyzed flavonols at 3 (1.7-fold) and 15 dpi (1.4-fold) (Table S2). The first significant increase in the amount of total analyzed flavonols was detected from 1 to 3 dpi. At 3 dpi, we also observed a significant increase in the susceptible compared to the resistant hop cultivar (Figure 4). Furthermore, we observed a 1.6-fold decrease of the total analyzed flavonols in the stems of the susceptible cultivar from 3 to 6 dpi, followed by a significant increase from 6 to 15 dpi (Figure 4). In the resistant cultivar, we did not detect any significant differences between the infected and control samples at any sampling time (Table S2). When comparing the amounts of total analyzed flavonols in the stem tissues between both cultivars during infection, we detected a significantly increased amount of total analyzed flavonols in the susceptible compared to in the resistant cultivar at 3, 6, 15, and 18 dpi (Figure 4).

Of all identified flavonols, the greatest portions of TAP in the susceptible cultivar were represented by kaempferol acetyl hexoside (4%), quercetin-3-rutinoside (3%), kaempferol acetyl rutinoside (3%), and kaempferol hexosyl rutinoside (3%); in the resistant cultivar, flavonols were represented by kaempferol acetyl rutinoside (5%), quercetin hexosyl dirhamnoside (3%), kaempferol hexosyl dirhamnoside (3%), and quercetin-3-glucoside (3%) (Figure 2). Individual flavonols were grouped in two subclasses, namely, quercetin glycosides and kaempferol glycosides.

In the infected stem samples of the susceptible cultivar, infection significantly increased the accumulation of kaempferol glycosides at 3 (1.7-fold), 15 (1.3-fold), and 18 dpi (1.3-fold), and quercetin glycosides at 3 (twofold) and 15 dpi (1.4-fold) (Table S2). In addition, significant increases in kaempferol glycosides and quercetin glycosides were observed from 1 to 3 dpi (Table S2.1). Compared to the resistant cultivar, the susceptible cultivar showed significant increases in kaempferol glycosides at 3, 6, and 15 dpi, and quercetin glycosides at 3, 15, and 18 dpi (Table S2.1). In the resistant cultivar, the amount of kaempferol glycosides steadily increased throughout the experiment, while a significant 1.4-fold decrease in quercetin glycosides was detected at 6 dpi in the infected compared to in the control stem samples, resulting in a significant decrease from 3 to 6 dpi (Table S2).

#### 2.2.3. Hydroxycinnamic Acids

**Figure 5:**
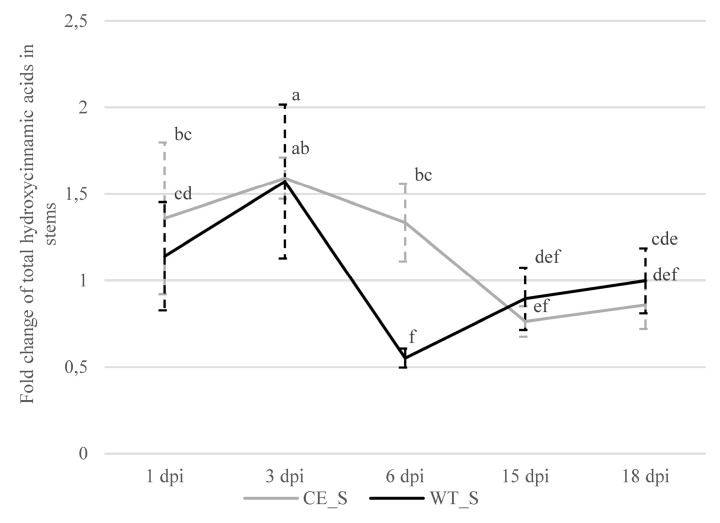
Fold changes (infected/control) of total analyzed hydroxycinnamic acids in the stems (S) of susceptible (CE; Celeia) and resistant (WT; Wye Target) hop cultivars. Data are presented as mean ± SD. Values below 1 indicate decreases in content compared to the control. Data were analyzed by one-way ANOVA and Duncan’s post hoc test. Different letters denote statistically significant differences at *p* ≤ 0.05.

In the infected compared to the control stem samples of the susceptible cultivar, we observed significantly higher amounts of total analyzed hydroxycinnamic acids at 1 (1.4-fold) and 3 dpi (1.6-fold), and a lower amount at 15 dpi (1.3-fold) (Table S2). Furthermore, in the stems of the susceptible cultivar, we observed a significant decrease in hydroxycinnamic acids from 6 to 15 dpi (Figure 5). In the resistant cultivar, we observed a significantly higher (1.6-fold) amount of hydroxycinnamic acids in the infected compared to the control samples at 3 dpi, and a significant increase was detected from 1 to 3 dpi (Figure 5). Furthermore, at 6 dpi, we observed a 1.8-fold lower amount of hydroxycinnamic acids in the infected compared to the control stems of the resistant cultivar, and a significant decrease was observed from 3 to 6 dpi (Figure 5).

Among hydroxycinnamic acids, *trans*-5-caffeoylquinic acid (1%), 3-*p*-coumaroylquinic acid (1%), feruloylquinic acid (0.5%), and 3-caffeoylquinic acid (0.5%) represented the greatest portions of TAP in the susceptible cultivar. However, in the resistant cultivar, feruloylquinic acid and *trans*-5-caffeoylquinic acid represented 1% of TAP, respectively, followed by caffeic acid sinapoyl hexoside and 3-*p*-coumaroylquinic acid, each accounting for 0.5% of TAP (Figure 2).

In the infected stem samples of the susceptible cultivar, we observed a significantly higher accumulation of caffeic acid sinapoyl hexoside at 3 (1.4-fold) and 18 dpi (1.8-fold), *cis*-5-caffeoylquinic acid at 18 dpi (2.5-fold), feruloylquinic acid at 3 (1.3-fold) and 6 dpi (1.4-fold), 3-caffeoylquinic acid at 3 dpi (2.4-fold), and 4-caffeoylquinic acid at 3 (1.3-fold) and 6 dpi (1.4-fold) (Table S3). In the infected stems of the resistant cultivar, we observed a significantly higher amount of *trans*-5-caffeoylquinic acid at 3 dpi (2.1-fold), 3-caffeoylquinic acid at 3 dpi (2.8-fold), 3-*p*-coumaroylquinic acid at 3 dpi (2.1-fold), and 5-*p*-cumaroylqunic acid at 1 dpi (2.6-fold) (Table S3). However, following an increase in the amount of hydroxycinnamic acids in the stems of the resistant cultivar at early sampling times, we observed lower amounts of feruloylqunic acid (1.6-fold), *trans*-5-caffeoylquinic acid (2.6-fold), 3-caffeoylquinic acid (2.2-fold), 3-*p*-coumaroylquinic acid (2.6-fold), and 5-*p*-cumaroylqunic acid (1.8-fold) at 6 dpi (Table S3).

When comparing individual hydroxycinnamic acids between the two cultivars, we observed significant increases in caffeic acid sinapoyl hexoside (1.5-fold), *cis*-5-caffeoylquinic acid (1.8-fold) and 5-*p*-coumaroylquinic acid (2.6-fold) at 1 dpi, *trans*-5-caffeoylquinic acid (2.1-fold) and 3-*p*-coumaroylquinic acid (2.1-fold) at 3 dpi, and a 1.1-fold increase in 4-*p*-coumaroylquinic acid at 15 dpi in the resistant cultivar. Furthermore, all detected hydroxycinnamic acids showed significant decreases in the resistant compared to the susceptible cultivar at 6 dpi (Table S3.1).

## 3. Discussion

*Verticillium* wilt is one of the main phytopathological issues of hop plantations, especially in Europe, where the first severe outbreaks of *Verticillium* wilt occurred [4,28]. Since the disease cannot be prevented by fungicides, the most effective method for *Verticillium* wilt management is the use of resistant and tolerant hop cultivars [29]. Although a large number of studies investigated resistance factors to *Verticillium* wilt [4,30,31,32,33], there is little information in the literature regarding the defense mechanisms underlying spatiotemporal changes in the phenolic content. In our study, the phenolic responses of the susceptible Celeia and the resistant Wye Target hop cultivars were monitored in root and stem tissues during the pathogenesis of *Verticillium* wilt.

The highest relative amount of fungal DNA in our study was detected at 1 dpi in the root tissue of both cultivars. The very high relative amount of fungi in the early sampling points of the root tissue can be explained on the basis of the observation of Zhang et al. [34] in cotton plants. The authors argued that, in early stages of infection, most fungal conidia that are attached to root surfaces and germ tubes start to penetrate the epidermis at 1 dpi, and about 80% of the conidia start to germinate and colonize the root tissue by 48 h postinoculation. By 4 dpi, the hyphal network is present on the root surface, and by 5 dpi, substantial colonization of the intercellular space of the root cortex and phloem can be observed. In the study of Zhang et al. [34], *V. dahliae* started to spread from the roots to the stem xylem vessels at 9 dpi, and we similarly observed the transition of *V. nonalfalfae* from roots to stems in the susceptible hop cultivar at 12 dpi, since the relative amount of fungi in the stems significantly increased from 6 to 12 dpi and root and stem tissues showed similar relative amounts of fungal DNA, i.e., 3.6-fold in roots and 1.2-fold in stems.

From this sampling point on, we detected increased colonization of roots in the susceptible but not in the resistant cultivar. Compared to the susceptible cultivar, the roots of the resistant cultivar in our experiment showed 1.48-fold lower relative fungal DNA amounts that decreased throughout the experiment, probably due to the defense responses of the plants, as also reported by Toueni et al. [29] and Cregeen et al. [30]. Smaller relative fungal DNA levels were also observed in resistant versus susceptible cultivars in other *Verticillium* host models, such as oilseed rape [35], cotton [34], lettuce [36], and olive [27]. 

Previous studies showed that hop plants react nonspecifically with the formation of physical barriers in the early stages of fungal colonization, e.g., the formation of tylose and the lignification of the endoderm layer in the roots of susceptible plants; in resistant hop plants, these processes are less intensive and delayed [22,23,30]. The results of our study suggested that infection with *V. nonalfalfae* at an early stage of pathogenesis causes the rapid synthesis of flavanols in the root tissues of resistant but not susceptible cultivars, which may help to limit the spread of the fungus, as observed in other plant species [37,38,39,40,41]. Accumulation of flavanols was also observed in response to infection with *V. dahliae* in the roots of a resistant cotton cultivar from 1 to 4 dpi [40]. Increased phenolic compounds were also observed in the roots of a resistant melon genotype (*Cucumis melo*) inoculated with *Fusarium oxysporum* f. sp. *melonis* race 1 [42]. 

Considering the stem tissue, the strongest response to infection was detected at 3 dpi, as we identified significantly increased contents of 14 and 9 IPCs in the susceptible and resistant cultivars, respectively. Studies performed on woody plants showed that flavanols contribute to physical defense against fungal pathogens. In poplar trees inoculated with biotrophic rust fungus (*Melampsora larici-populina*), higher amounts of catechin and proanthocyanidins (PAs) were determined at the site of the fungal infection, inhibiting the germination of rust spores and reduced hyphal growth [37]. Furthermore, the accumulation of flavanols and PAs in the spruce bark of Norway spruce saplings inhibited growth of the fungal pathogen *Ceratocystis polonica* after inoculation [38]. In cotton, these molecules create chemical barriers in wood and prevent pathogenic spread [39,40]. Higher flavanol contents were detected in obstructive vascular formations, such as tyloses and gels, at 4 dpi in *Platanus* × *acerifolia* seedlings inoculated with *Ceratocystis firnbriata* f.sp. *platani* [41]. Similarly, Cregeen et al. [30] observed extensive tylose formation around vessels in the stems of a susceptible hop cultivar as an early response to infection with *V. nonalfalfae*. Our results suggested that individual flavanols accumulated more extensively in the stems of the susceptible cultivar compared to those of the resistant, which, with tylose formation, may represent a defense response against *V. nonalfalfae*. However, these processes can lead to the complete sealing of xylem vessels, resulting in drought stress, reduced plant vitality, and promotion of successful fungal infection [7].

In the late stage of infection in the stems of the susceptible cultivar, we observed decreased flavanols and hydroxycinnamic acids at 15 dpi. In the resistant cultivar, however, decreases in flavanols, hydroxycinnamic acids, and quercetin glycosides were observed at 6 dpi. A similar pattern of flavanol accumulation during the early stage of infection and a rapid decrease to 7 dpi were also observed in *Platanus* × *acerifolia* inoculated with *Ceratocystis fimbriata* f.sp. *platani* [41]. Similarly, Mikulic-Petkovsek et al. [43] observed a 2.7- to 3.8-fold decrease in the content of total quercetin glycosides in raspberry cultivars infected with *Leptosphaeria coniothyrium* compared to healthy cane tissue. The authors argued that the decrease may have been due to the use of quercetin glycosides in the synthesis of flavanols in the phenylpropanoid pathway [43]. In the case of the *Verticillium* host model, the decrease in quercetin glycosides could also be due to the hydrolyzing action of the fungus on these compounds [44]. In this way, the fungus suppresses defense-related quercetin glycosides, e.g., rutin, and overcomes the host defense mechanisms [18].

Our results were also consistent with studies reporting that the interaction between the pathogen and the host often alters the amount of different hydroxycinnamic acids [17,45,46]. Decreased hydroxycinnamic acids may be due to their role and consumption in the synthesis of complex cell-wall-bound phenolics, e.g., lignin and suberin [47]. Thus, in both hop cultivars, the decreases in the amount of hydroxycinnamic acids in the infected stem tissues could may have coincided with cell-wall lignification or fortification; in the resistant cultivar, an earlier response to fungal pathogen invasion was observed, since we detected a 1.8-fold lower amount of hydroxycinnamic acids in the infected compared to the control samples at 6 dpi, and a 2.8-fold decrease from 3 to 6 dpi (Figure 5). In the susceptible cultivar, however, we observed a 1.6-fold higher amount of hydroxycinnamic acids in the infected compared to the control samples at 3 dpi, and a gradual decrease from 3 to 15 dpi (Figure 5). The delayed decrease in the amount of hydroxycinnamic acids and consequently the probable delayed cell-wall reinforcement in the susceptible cultivar might be due to the suppression of lignification caused by the pathogen [48,49]. An increase in cell-wall-bound phenolics at the early stage, followed by a decrease during the midstage of infection were also observed in cell cultures of tomatoes (*Lycopersicon esculentum* Mill.) infected by *V. nonalfalfae* [20] and in potatoes infected by *V. dahliae* [18]. Similarly, following a decrease in cell-wall-bound phenolics, lignin deposition was observed after 96 h postinoculation in *Sorghum vulgare* Pers. cv. M.P. inoculated with *Sclerotium rolfsii* Sacc [50]. Additionally, the resistance of broccoli to *V. dahliae* is associated with higher contents of lignin and cell-wall-bound phenolics [51].

Besides phenolics, there may be other defense mechanisms that contribute to reduced colonization of the fungus in hop plants that were described in other plant species, e.g., triterpenoid saponin avenacin A-1, which plays an important role in protecting the roots of oats against the fungal pathogen *Gaeumannomyces graminis* var. *tritici* [52], 3-deoxyanthocyanidin flavonoids, which accumulate in sorghum in response to infection with *Colletotrichum graminicola* [53], and elemental sulphur (S8), for which antimicrobial and antifungal properties are well known [54]. Recent studies found an increased concentration of elemental sulphur in representative species of Solanaceae (tomato, tobacco), Leguminosae (French bean), and Malvaceae (cotton) in response to vascular phytopathogens [55,56].

## 4. Materials and Methods

### 4.1. Plant Material

Plant inoculation was performed according to the protocol described by [57]. Briefly, one-year-old rooted cuttings of *Verticillium*-wilt-resistant (WT; Wye Target) and -susceptible (CE; Celeia) hop cultivars were artificially inoculated by immersing the roots in a conidial suspension (5 × 10^6^ conidia per mL) of *V. nonalfalfae* lethal (PV1) pathotype (Isolate T2) for 10 min, while the control plants were mock-inoculated using sterile water. Afterward, the plants were repotted to a sterile commercial substrate and grown in a growth chamber under controlled conditions [57].

*Verticillium*- and mock-inoculated hop plants of each cultivar were sampled at 1, 3, 6, 12, 15, 18, and 30 days postinoculation (dpi). Fifteen inoculated and three mock-inoculated hop plants (stems and roots) of each cultivar were used for fungal colonization analysis; three mock-inoculated and eight *Verticillium*-inoculated stem or root samples from 1, 3, 6, 15, and 18 dpi were used to analyze the phenolic compounds. On the basis of a similar relative amount of fungal DNA present in the inoculated samples, eight root or stem samples of *Verticillium*-inoculated hop plants were pooled into four inoculated root or stem samples and used for further analysis of phenolic compounds.

Samples were taken from the washed roots and stems; bines were cut at the first node. Sampled tissue was freeze-dried with liquid nitrogen, ground to a fine powder using autoclaved mortars and pestles, and stored at –80 °C until isolation of total genomic DNA and extraction of phenolic compounds.

### 4.2. V. nonalfalfae qPCR Quantification

Total genomic DNA was extracted from the inoculated and control samples as described by Kump and Javornik [58]. The concentration of DNA isolates was determined by means of spectrophotometry and diluted to 25 ng µL^−1^. Relative quantity of *V. nonalfalfae* DNA in plants was determined using Applied Biosystems^®^ 7500 real-time quantitative PCR system (Applied Biosystems, Foster City, USA) and Fast SYBR^®^ Green technology (Thermo Fisher Scientific). Fungal DNA levels were detected and estimated with *V. nonalfalfae* lethal genotype (PG2)-specific primer pair 5-1gs-F-GAGCGGGTCGATACGATTCA and 5-1gs-R-GGTGATGTCCAGCACAGTGATAC. The absence of fungal DNA in the control samples was also confirmed by real-time PCR. Real-time PCR was carried out in a 10 µL total reaction volume containing 5 µL Fast SYBR Green Master Mix, 25 ng of total genomic DNA, and 300 nM of each *V. nonalfalfae* genotype-specific forward and reverse primer.

The following amplification program was used: 95 °C for 20 s, 40 cycles at 95 °C for 3 s, and 60 °C for 30 s. This was followed by the melting curve stage at 95 °C for 15 s, 60 °C for 60 s, 95 °C for 15 s, and 60 °C for 15 s to monitor primer specificity and the formation of nonspecific PCR amplicons.

All samples were amplified in two technical replicates acquired from the same total DNA sample. The amplification levels of the target sequence were determined as cycle-threshold (Ct) values that represent the number of cycles needed to reach a threshold of amplification fixed in the exponential phase of the PCR reaction [59]. The amplification efficiency of the amplified target sequence (5-1gs) was determined from a standard curve obtained from five 5-fold serial dilutions of all per-dpi-pooled total genomic DNA samples ranging from 25 to 0.04 ng µL^−1^. Normalization of relative fungal DNA quantity in plant samples was performed by using the plant reference gene DRH1 (F- 5’-CCAACCTACTGGGCTTCGAC-3’ and R- 5’-CAGAATGGGTATGATCGGGC-3’), as previously described by Stajner et al. [60], and relative changes in the quantity of fungal DNA were determined using the 2^−ΔCt^ method [61]. Specific primer pairs that amplified the locus of the lethal pathotype of *V. nonalfalfae* (PG2 5-1gs) were optimized to verify successful colonization and to assess the relative amount of fungal DNA in hop plants.

### 4.3. Extraction and Determination of Phenolic Compounds Using HPLC–Diode-Array Detector (DAD)–MS^n^ Analysis

Root- and stem-tissue samples of both cultivars were used for the extraction and determination of phenolic compound contents. Fine tissue powders (200 mg) of the control or infected samples were mixed with 1.5 mL of 80% methanol and homogenized in a cooled ultrasonic bath for 30 min. Extracts were centrifuged at 10,000 rpm for 10 min at 4 °C and filtered through a 0.20 μm membrane filter (Macherey-Nagel, Düren, Germany) in vials before injection into the HPLC system. 

The used standards and HPLC system conditions for phenolic compound quantification were the same as those described by Mikulic-Petkovsek et al. [62]. Phenolic compounds were analyzed using a Thermo Finnigan Surveyor HPLC system (Thermo Scientific, San Jose, CA, USA) with a diode-array detector (DAD) at 280 and 350 nm. Flavanols (epicatechin, catechin, procyanidins, etc.) and hydroxycinnamic acid derivatives (chlorogenic, *p*-coumaric, ferulic, caffeic acid, etc.) were detected at 280 nm, whereas flavonol derivatives (kaempferol, quercetin, etc.) were detected at 350 nm. Phenolic compounds were identified by comparison of UV–vis spectra and the retention times of phenolics and standards, and confirmed with a mass spectrometer (Thermo Scientific, LCQ Deca XP MAX, Waltham, USA) with electrospray ionization (ESI) operating in negative ion mode. Mass-spectrometer conditions were the same as those reported by Mikulic-Petkovsek et al. [13]. Concentrations of phenolic compounds were calculated from the peak areas and expressed as µg g^−1^ fresh weight (FW).

### 4.4. Statistical Analysis

Data were analyzed using an R software environment for statistical computing [63] and R package agricolae [64].

One-way ANOVA and Duncan’s post hoc test were used to analyze differences in the relative amounts of *V. nonalfalfae* DNA (ΔCt) between the root samples of both cultivars at different sampling times. To analyze differences in the relative amounts of fungal DNA (ΔCt) between stem samples, the Kruskal–Wallis rank-sum test and the Holm–Bonferroni method were performed, since the data did not meet the assumption of normality and homogeneity of the variance. Statistically significant differences between root and stem samples were tested at *p* ≤ 0.05.

One-way ANOVA was performed to determine the significance of *V. nonalfalfae* infection on individual phenolic compounds and on the total amount of each phenolic class (flavanols, flavonols, and hydroxycinnamic acids) in the root and stem tissues, respectively. Differences in the amounts of each phenolic compound between samples were tested by Duncan’s test at the significance level of *p* ≤ 0.05. Results were presented as the average content of individual phenolic compounds per 1 g of fresh-weight sample (µg g^–1^ FW) with standard deviation (SD). Different letters denote significant differences at *p* ≤ 0.05 (Tables S1–S3). To compare the phenolic contents among sampling times and cultivars, infected samples were normalized to the corresponding controls, and one-way ANOVA and Duncan’s test (*p* ≤ 0.05) were performed (Tables S1.1, S2.1 and S3.1).

## 5. Conclusions

The pattern of the phenolic compounds (individual and total) in the roots showed that there were no differences between the control and infected samples in the susceptible cultivar; in the resistant cultivar, a significant increase in total flavanols was observed at 3 dpi in the infected compared to the control root samples. This suggested that flavanols may be involved in defense responses in the roots of the resistant cultivar, thereby helping to prevent infection.

In response to infection in the stems, the contents of six different individual flavanols, four flavonols, and six hydroxycinnamic acids changed between sampling times in both cultivars. Considering temporal changes in the amount of phenolic compounds, we observed less extensive accumulation in the stem tissue of the resistant cultivar, since flavanols and hydroxycinnamic acids accumulated only at 3 dpi. However, in the susceptible cultivar, we observed accumulation of phenolic compounds in additional sampling points, namely, flavanols at 1 and 3 dpi, flavonols at 3 and 15 dpi, and hydroxycinnamic acids at 1 and 3 dpi. Additionally, in comparison with the resistant cultivar, the susceptible cultivar showed significant increases in flavonols in the stems at 3 and 15 dpi and hydroxycinnamic acids at 6 dpi.

As already mentioned, the extensive accumulation of cell-wall-bound phenolic compounds in the stems of the susceptible cultivar could lead to a complete sealing of the xylem vessels and to drought stress, which may contribute to the death of susceptible hop plants. 

These data suggested that phenolic compounds might be involved in the defense reactions of hop plants against *V. nonalfalfae*, especially in the roots of resistant and in the stems of susceptible hop cultivars. The results of our study substantiate the interest to investigate the mode of action of individual phenolic compounds that accumulate differently during the pathogenesis of *Verticillium* wilt.

## Supplementary Materials

The following are available online at https://www.mdpi.com/2223-7747/9/7/841/s1. Table S1: Amount (µg/g) of flavanols in the roots (R) and stems (S) of susceptible (CE; Celeia) and resistant (WT; Wye Target) hop cultivars; Table S1.1: Fold changes (infected/control) of flavanols in the roots (R) and stems (S) of susceptible (CE; Celeia) and resistant (WT; Wye Target) hop cultivars; Table S2: Amount (µg/g) of flavonol derivatives in the stem tissues of susceptible (CE-S; Celeia stems) and resistant (WT-S; Wye Target stems) hop cultivars; Table S2.1: Fold changes (infected/control) of flavonol derivatives in the stems (S) of susceptible (CE; Celeia) and resistant (WT; Wye Target) hop cultivars; Table S3: Amount (µg/g) of hydroxycinnamic acids in the stem tissues of susceptible (CE-S; Celeia stems) and resistant (WT-S; Wye Target stems) hop cultivars; Table S3.1: Fold changes (infected/control) of analyzed hydroxycinnamic acids in the stems (S) of susceptible (CE; Celeia) and resistant (WT; Wye Target) hop cultivars.
